# The evaluation of microbiology and prognosis of fournier’s gangrene in past five years

**DOI:** 10.1186/s40064-014-0783-8

**Published:** 2015-01-13

**Authors:** Lap-Ming Tang, Yu-Jang Su, Yen-Chun Lai

**Affiliations:** Department of Emergency Medicine, Mackay Memorial Hospital, Taipei, Taiwan; Department of Oral Hygiene, College of Oral Medicine, Taipei Medical University, Taipei, Taiwan; Department of Anesthesiology, Taiwan Adventist Hospital, Taipei, Taiwan

**Keywords:** Escherichia coli, Fournier’s Gangrene, Microbiology, Poly-microbial, Prognosis

## Abstract

**Objectives:**

Fournier’s gangrene (FG) is an devastating disease that affects the perineum and genitourinary region, and is commonly a result of poly-microbial infection. This study is aimed to determine the correlation between micrology and prognosis of FG in the past five years.

**Methods:**

This study was a retrospective cohort study that was designed to study the trends in micrology and prognosis of FG. From the PubMed database, articles published in the recent 5 years (from Jan1^st^, 2009 to Dec 31^st^, 2013) were reviewed. A total of 19 articles (each with n > 30 and with thorough data descriptions in the topic of Fournier's gangrene), were enrolled in this study. The consolidated data was further analyzed by commercial statistical software (SPSS for Windows).

**Results:**

The twenty-two studies have covered FG cases from year 1981 to 2011, with a mean duration of 9.2 years. The total number of cases is 4,365. Majority of the cases are male (84.1%). The mean age and mortality rate is 51.8 ± 5 years old and 11.1 ± 8.9%, respectivly. The most commonly found pathogen is poly-microbial organism (54%), followed by *Escherichia coli* (46.6%) and *Streptococcus* (36.8%). The major risk factors are diabetes (43.7%), Body mass index of > 30 (40.7%), and hypertension (38.1%). Mortality rate in older patient group (age > 51.8 years old) is significantly higher than those of the younger group (22% vs. 5.5%, *p* = *0.0001*).

**Conclusions:**

Older patients with genital or perineal pain should be examined for crepitus dermis. When a patient is diagnosed with FG, swift consultation with surgeons and administration of broad-spectrum antibiotics are required in order to save the patient’s live.

**Electronic supplementary material:**

The online version of this article (doi:10.1186/s40064-014-0783-8) contains supplementary material, which is available to authorized users.

## Background

Fournier’s gangrene (FG) is a devastating necrotising disease that affects the perineum and genitourinary regions. The common cause of FG is poly-microbial infections, where the diabetes mellitus is an attributing common risk factor (Shyam and Rapsang [2013]). Study has shown that males, especially in their 60 to 70s, are more often affected by FG when compared to other populations (Rodríguez Alonso et al. [2000]). Aside from diabetes, other risk factors of FG also include chronic alcoholism, renal failure, and obesity (Montoya Chinchilla et al. [2009]). The majorities of FG studies have shown that early diagnosis and aggressive management of FG are required to significantly improve patient outcome. Due to the fact that FG is not a common disease, a prospective study is difficult to perform. Therefore, In this study, a large number of FG cases that have occurred in the past five years are gathered by retrospective literature review and analyzed to determined the relationship between micrology and prognosis of FG.

## Methods

A retrospective cohort study was designed to investigate the correlation between micrology and prognosis of FG. A search from the PubMed database returned a total of 1,015 literatures that contain the keyword “Fournier’s gangrene”. The resulting literatures were further limited to the literature s that were published within the past 5 years, (from Jan.1, 2009 to Dec 31, 2013), where a total of 330 articles remained. The articles that contain case numbers of less than 30 were excluded from the study, since the low sample number cannot establish a normal distribution for the evaluation of statistical significance. In the end, there were 19 articles that was enrolled into this study. The combined data and descriptions of Fournier’s gangrene are listed in Table. 1 References (Martinschek et al. [2012]) to (Ersoz et al. [2012]). These enrolled research literatures are from Germany (n = 4, 21%), Turkey (n = 4), United States of America (n = 2), Pakistan (n = 2), Spain (n = 2), Mexico (n = 1), Brazil (n = 1), Taiwan (n = 1), Tunisia (n = 1) and Croatia (n = 1). The data was analyzed with a commercial statistical software (SPSS for Windows, version 11.0, SPSS Ltd., Chicago, IL). Statistical χ2 tests were performed and the significance was set at a p value of less than 0.05 (2-tailed).

**Table 1:** **There were 19 articles (from January 1, 2009 to December 31, 2013) enrolled into this study**

Duration	Duration [years]	First author	Country	Number	Male %	Female %	Mean age	Mortality rate %
1981 ~ 2010	29	Martinschek et al. ([2012])	Germany	55	61.8	38.2	48	16.4
1994 ~ 2006	12	Macro et. al. ([2010])	Spain	51	94	6	63	16
1995 ~ 2010	15	Sallami et a. ([2012])	Tunisia	40	NA	NA	52.8	17.5
1995 ~ 2010	15	Altarac et al. ([2012])	Croatia	41	95	5	62	36.6
1996 ~ 2006	10	Yilmazlar et al. ([2010])	Turkey	80	71.3	28.7	57	21
1996 ~ 2006	10	Ozturk et al. ([2011])	Turkey	44	52.3	47.7	57	36.3
1996 ~ 2008	12	Czymek et al. ([2010])	Germany	38	NA	NA	57.7	21.1
1998 ~ 2006	8	MEHL et al. ([2010])	Brazil	40	77	23	47.2	20
1999 ~ 2009	10	Koukouras et al. ([2011])	Germany	45	88.9	11.1	50	15.6
2000 ~ 2008	8	Malik et al. ([2010])	Pakistan	73	91.8	8.2	57.3	17.8
2000 ~ 2008	8	Chen et al. ([2011])	Taiwan	50	NA	NA	53.6	12
2001 ~ 2004	4	Sorensen et al. ([2009])	USA	1680	97.7	2.3	50.9	7.5
2001 ~ 2011	10	Roghmann et al. ([2012])	Germany	44	NA	NA	59	30
2002 ~ 2007	5	Morua et al. ([2009])	Mexico	63	96	4	47.5	12
2002 ~ 2007	5	Ullah et al. ([2009])	Pakistan	60	83.3	16.7	47	7
2003 ~ 2008	5	Vargas et al. ([2011])	Spain	42	100	0	51	17
2004 ~ 2010	36	GÖKTAŞ et al. ([2012])	Turkey	36	NA	NA	55.5	11.1
2004 ~ 2011	7	Bjurlin et al. ([2013])	USA	122	97.6	2.4	49	4.9
2005 ~ 2008	3	Ersoz et al. ([2012])	Turkey	52	69	31	55	23.8

The combined data and descriptions of Fournier’s gangrene are listed in Table 1.

NA = Not Available.

## Results

The twenty-two studies have covered FG cases from year 1981 to 2011, with a mean duration of 9.2 years. The total number of cases is 2,656. Majority of cases are male (84.1%) and female is accounted for 15.9%.

**Figure 1:**
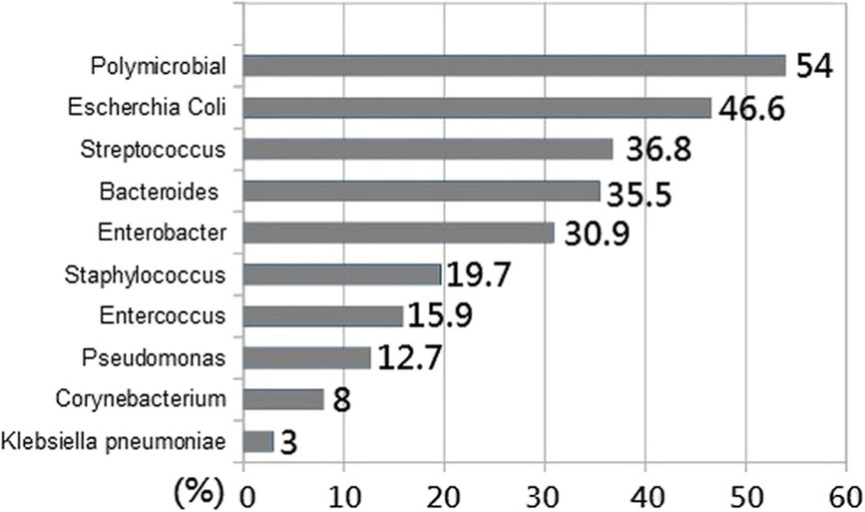
**List of commonly found pathogens involving Fournier’** **s gangrene** **(presented in percentages).**

The mean age of the patients is 51.8 years old, and the average mortality rate is 11.1 ± 8.9%. When comparing between the older age group (age > 51.8 years old) and the younger group (age of less or equal to 51.8), the mortality rate was found to be higher in the older group than younger (22 ± 8.8% versus 5.5 ± 2%, *p* = *0.0001*) The most commonly found pathogen is poly-microbial organism (54%), followed by *Escherichia coli* (46.6%) and *Streptococcus* (36.8%). The other contributing pathogens also include *Bacteroides*, *Enterbacter*, *Staphylococcus*, *Enterococcus*, *Pseudomonas*, *Corynebacterium*, and *Klebsiella pneumoniae* (Figure 1).

**Figure 2:**
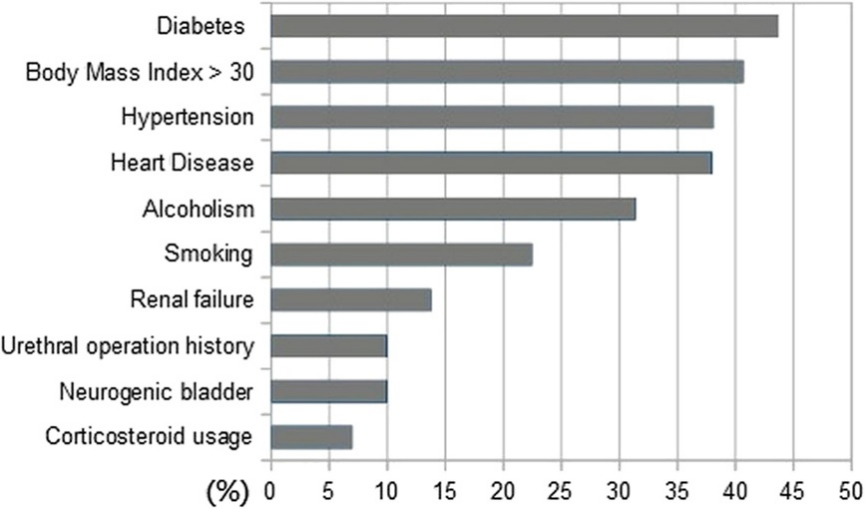
**List of commonly found risk factors involving Fournier’** **s gangrene** **(presented in percentages).**

The major risk factors for FG are diabetes (43.7%), body mass index of higher than 30 (40.7%), and hypertension (38.1%). Other risk factors also include heart disease (38%), alcoholism (31.4%), smoking (22.5%), renal failure (13.8%), urethral operation history, neurogenic bladder, and corticosteroid user (Figure 2).

## Discussion

Fournier’s gangrene (FG) is a rare emergent condition that affects the perineum and urogenital region. The clinical course of FG is fulminant and serious regardless of parenteral antibiotic treatment. The median time from syptom presentations to skin gangrenous change is 6 days (Altarac et al. [2012]). When managing FG patients, this gangrenous tissue requires extensive and repeated debridement (Sallami et al. [2012]). Several literatures have shown that patients with diabetes, old age, low blood pressure, high creatine kinase, high lactate, abdominal affection, hemoglobin of less than 10 g/dL, and platelet count of less than 150 × 10^9^/L are associated with poor outcomes (Martinschek et al. [2012]; Ruiz-Tovar et al. [2012]).

Many literatures have determined that the risk factors of FG include diabetes mellitus, hypertension, heart disease, smoking, long-term steroid therapy, alcoholism or alcohol abuse, in hot and humid season, and renal failure (Martinschek et al. [2012]; Sallami et al. [2012]; Czymek et al. [2010]; Mehl et al. [2010]; Malik et al. [2010]; Ullah and Khan [2009]). Out of the many risk factors, diabetes mellitus is still the highest influencing factor on FG where 43.7% of FG patients are diabetic. A report by Czymek et. al. showed that being overweight is also a risk factor of FG, where nearly 40 % of FG patients have body mass indexes (BMIs) of higher than 30 (Czymek et al. [2010]; Mehl et al. [2010]). Although there are several known risk factors that can lead to the development of FG, the clinical onset of FG is still unpredictable.

The most common symptoms of FG are perineal pain and fever that are accompanied by swelling and reddening of perineum or genital area, and the gangreneous change of overlaying skin (Ruiz-Tovar et al. [2012]).

The most common microbiology involved in FG is poly-microbial infection (54%), and the most common found pathogen isolate is *Escherichia coli* (46.6%). Others contributing pathogen are *Streptococcal infection*, *Bacteroides*, *Enterobacter*, *Staphylococcus*, *Enterococcus*, *Pseudomonas*, *Corynebacterium*, *and Klebsiella pneumoniae* (Rodríguez Alonso et al. [2000]; Czymek et al. [2010]; Mehl et al. [2010]). Broad-spectrum antibiotic treatment is suggested to adequately cover poly-microbial pathogen, and careful patient monitoring is required to avoid is fungal or hospital-acquired pathogen infection (Bjurlin et al. [2013]).

In terms of gender, there was no significant mortality rate that was found between the genders (Ersoz et al. [2012]). A study from Spain (n = 51) showed that the survivors of FG are 13.5 years younger than those who have died (60 versus 73.5, *p* = *0.02*) (Luján Marco et al. [2010]). In a 2012 report from Turkey ( n = 52), the non-survivors group are older in age than survivors (62 versus 55 years old]. In our study, the results also showed that the older patients age had higher rates of mortality. This result is concurrent with in the other previous studies, where increased age was shown to be related to higher mortality rate (Martinschek et al. [2012]; Roghmann et al. [2012]).

Although FG is rare, its rapid progression can lead to life-threatening conditions that require early surgical intervention and parenteral antibiotics to improve patient outcomes (Morua et al. [2009]). The mortality rate of FG remains high and ranges from 4.9 to 36.6% in the recent five years. Due to its high mortality rate and rapid progress, FG must be regarded in clinical settings. The reduction of obesity, alcohol consumption, tobacco use is helpful in reducing the possible risk of FG. Furthermore, older patients with genital or perineal pain should be examined for crepitus dermis. Finally, when a patient is diagnosed with FG, swift consultation with surgeons and administration of broad-spectrum antibiotics are required in order to save the patient’s live.

## Authors’ information

Dr. Lap-Ming Tang and Dr. Yu-Jang Su are senior attending physicians worked at Department of Emergency Medicine, Mackay Memorial Hospital, Taipei 10449, Taiwan. Dr. Yu-Jang Su is also awarded six times of best teacher of Mackay Memorial Hospital, Taipei till 2014, and teaches at department of Oral Hygiene, College of Oral Medicine, Taipei Medical University, Taipei, Taiwan as an Assistant Professor. Dr. Yen-Chun Lai is a professional anesthesiologist working at Department of Anesthesiology, Taiwan Adventist Hospital, Taipei, Taiwan. We all have copious clinical experiences in practice.

## Authors’ original submitted files for images

Below are the links to the authors’ original submitted files for images. Authors’ original file for figure 1 Authors’ original file for figure 2
