# Supplementary figures and images for: The evaluation of microbiology and prognosis of fournier’s gangrene in past five years
Source: Springerplus. 2015 Jan 13;4(1):14. doi: 10.1186/s40064-014-0783-8 (PMC4305518; doi:10.1186/s40064-014-0783-8)

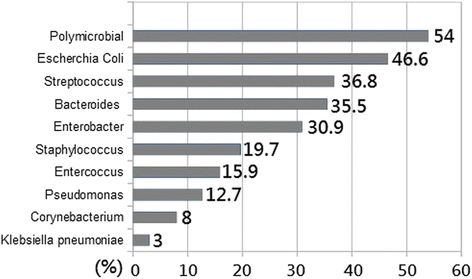

Supplement: Supplementary file 1 — Authors’ original file for figure 1 [file 40064_2014_783_MOESM1_ESM.gif]

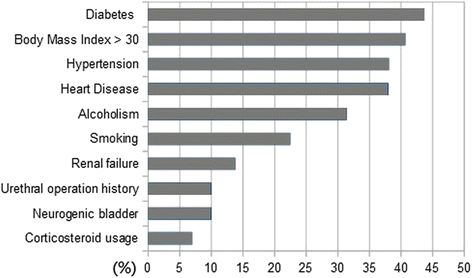

Supplement: Supplementary file 2 — Authors’ original file for figure 2 [file 40064_2014_783_MOESM2_ESM.gif]
